# Supplementary material for: Immunoinformatics analysis of candidate proteins for controlling bovine paratuberculosis
Source: PLoS One. 2022 Nov 21;17(11):e0277751. doi: 10.1371/journal.pone.0277751 (PMC9678287; doi:10.1371/journal.pone.0277751)
Supplement: S1 Table — (DOCX) [file pone.0277751.s001.docx]

**Supporting Information**

**Immunoinformatics Analysis of Candidate Proteins for Controlling Bovine Paratuberculosis**

Maryam Sadat Moezzi^1^, Abdollah Derakhshandeh^1^, Farhid Hemmatzadeh^2^

^1^Department of Pathobiology, School of Veterinary Medicine, Shiraz University, Shiraz, Iran.

^2^School of Animal and Veterinary Sciences, The University of Adelaide, South Australia, Australia.

**S1 Table.** IFN-γ inducer HTL epitopes

| ht-MAP2191 | ht-FAP-P |
| --- | --- |
| KLGPTLDRLNSVAAM  LGPTLDRLNSVAAML  GPTLDRLNSVAAMLE  PTLDRLNSVAAMLEK  TLDRLNSVAAMLEKN  LDRLNSVAAMLEKNR  VAAMLEKNRDNLSKA  AAMLEKNRDNLSKAL  KNRDNLSKALPGLKK  NRDNLSKALPGLKKF  RDNLSKALPGLKKFE  DNLSKALPGLKKFEI  NLSKALPGLKKFEIT  LSKALPGLKKFEITS  LPGLKKFEITSGESV  PGLKKFEITSGESVS  GLKKFEITSGESVSN  LKKFEITSGESVSNG  KKFEITSGESVSNGF  SNGFYYNAFVPNLAI  NGFYYNAFVPNLAIP  GFYYNAFVPNLAIPE  FYYNAFVPNLAIPEL  YYNAFVPNLAIPELI  YNAFVPNLAIPELIQ  NAFVPNLAIPELIQP  AFVPNLAIPELIQPF  YYFGFRRNDPNMPRA  YFGFRRNDPNMPRAL  FGFRRNDPNMPRALF  GFRRNDPNMPRALFP  FRRNDPNMPRALFPW | QPPPVANDTRIVMGR  DTRIVMGRLDQKLYA  TRIVMGRLDQKLYAS  DQKLYASAEANNAKA  QKLYASAEANNAKAA  KLYASAEANNAKAAV  PGTRINQDSTPLNGA  GTRINQDSTPLNGAN |
