# Supplementary material for: Immunoinformatics analysis of candidate proteins for controlling bovine paratuberculosis
Source: PLoS One. 2022 Nov 21;17(11):e0277751. doi: 10.1371/journal.pone.0277751 (PMC9678287; doi:10.1371/journal.pone.0277751)
Supplement: S1 Fig — (DOCX) [file pone.0277751.s002.docx]

**Supporting Information**

**Immunoinformatics Analysis of Candidate Proteins for Controlling Bovine Paratuberculosis**

Maryam Sadat Moezzi^1^, Abdollah Derakhshandeh^1^, Farhid Hemmatzadeh^2^

^1^Department of Pathobiology, School of Veterinary Medicine, Shiraz University, Shiraz, Iran.

^2^School of Animal and Veterinary Sciences, The University of Adelaide, South Australia, Australia.


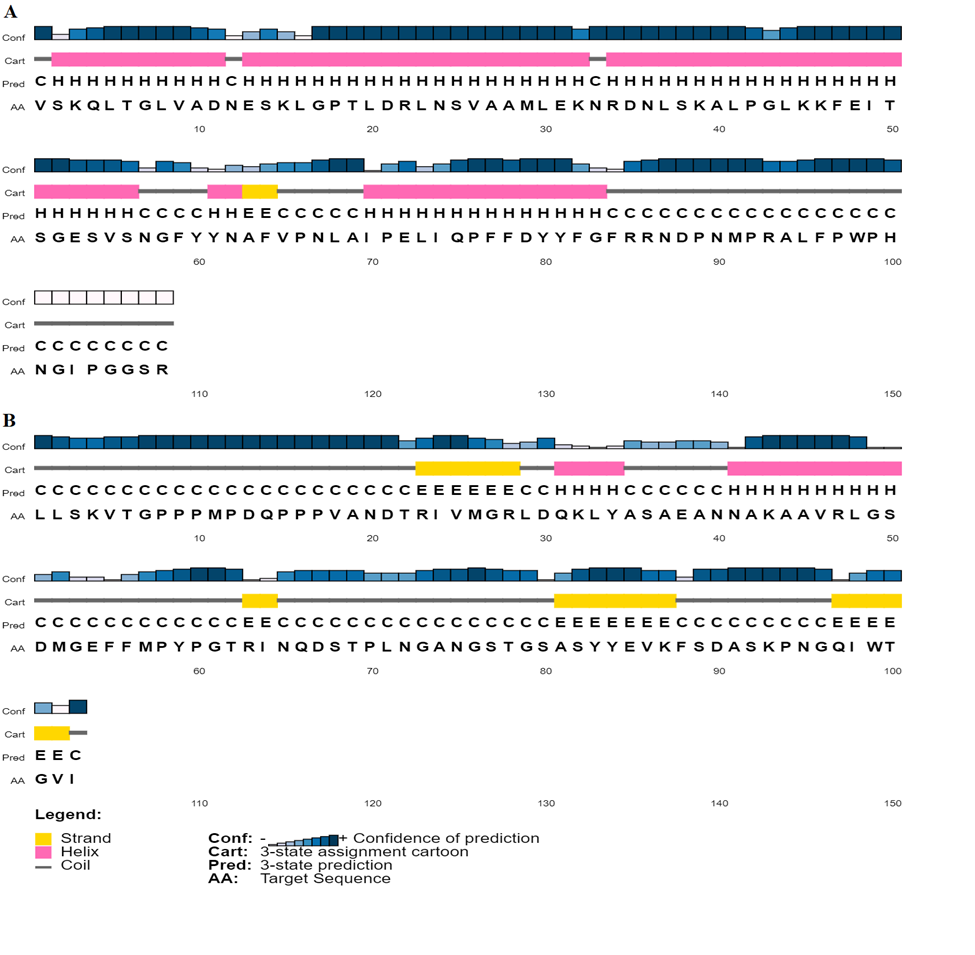


**S1A Fig.** Predicted secondary structure of A) ht-MAP2191 and B) ht-FAP-P


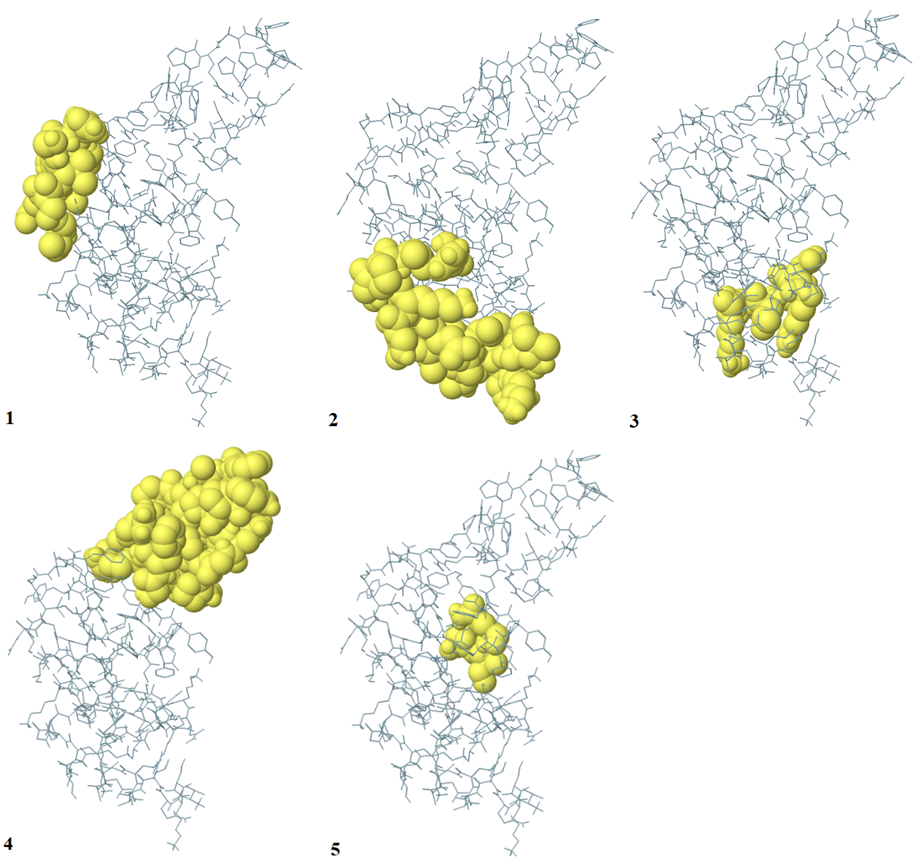


**S1B Fig.** Discontinuous epitopes on the ht-MAP2191 construct’s 3D structure. The gray sticks and the yellow surface indicate the protein construct and conformational B-cell epitopes, respectively.


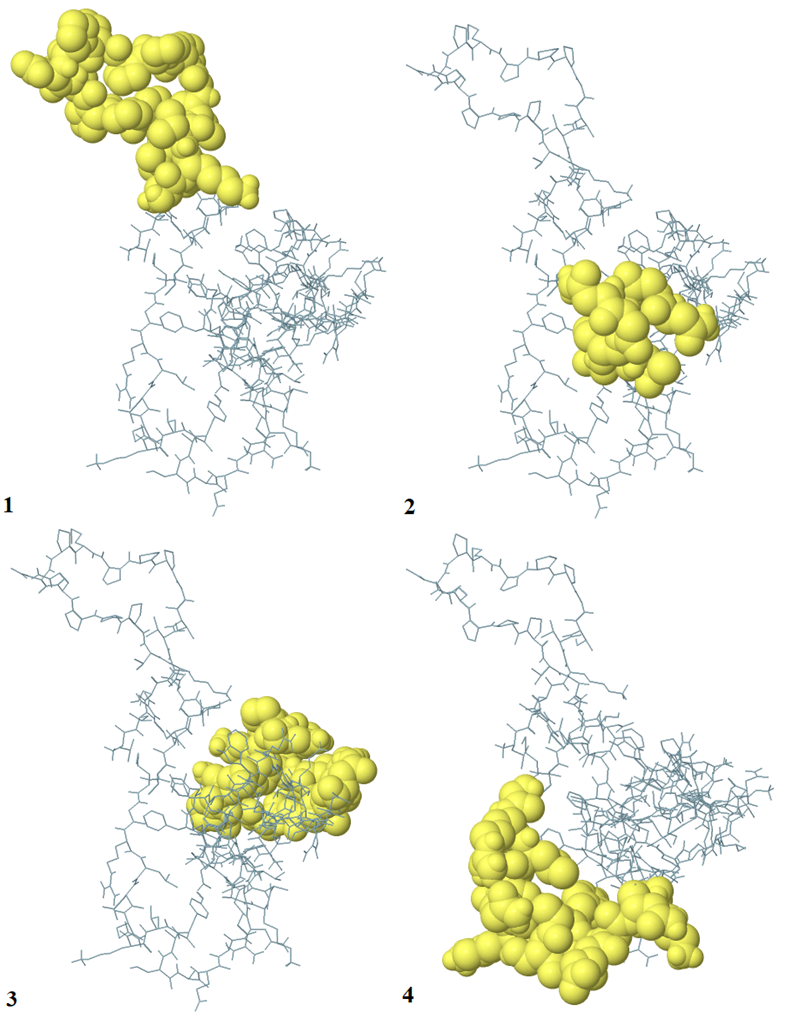


**S1C Fig.** Discontinuous epitopes on the ht-FAP-P construct’s 3D structure. The gray sticks and the yellow surface indicate the protein construct and conformational B-cell epitopes, respectively.
