# Supplementary material for: Immunoinformatics analysis of candidate proteins for controlling bovine paratuberculosis
Source: PLoS One. 2022 Nov 21;17(11):e0277751. doi: 10.1371/journal.pone.0277751 (PMC9678287; doi:10.1371/journal.pone.0277751)
Supplement: Attachment — Submitted filename: Manuscript Comments.docx [file pone.0277751.s003.docx]

I have reviewed the manuscript entitled  "Immunoinformatics Analysis of Candidate Proteins for Controlling Bovine Paratuberculosis" by Moezzi et. al., The authors have evaluated the efficacy of two proteins MAP2191 and FAP-P to be a potent vaccine candidate against MAP infection using immunoinformatics approaches.

I have the following comments regarding the manuscript.

1. The authors have selected only one proteins MAP2191 out of cluster of 6 genes, why only one?, it has been mentioned that all these proteins are involved MAP invasion, survival and virulence (Hemati Z, 2018; Semret M, 2005).
2. The author did not mention anywhere whether these protein will act as protective antigen against all serovars or single serovars of MAP.
3. The author should also explore the Virulence database.
4. The author can explore the confident B-Cell epitope by using two or more different tool and common prediction by all these will be considered confident B-cell epitope (conformational as well as linear epitope).
