# Supplementary material for: Immunoinformatics analysis of candidate proteins for controlling bovine paratuberculosis
Source: PLoS One. 2022 Nov 21;17(11):e0277751. doi: 10.1371/journal.pone.0277751 (PMC9678287; doi:10.1371/journal.pone.0277751)
Supplement: Attachment — Submitted filename: Response to reviewer.docx [file pone.0277751.s004.docx]

**Editor in Chief,**

**PLOS ONE**,

**Dear Sir/Madam**

We really appreciate reviewer comments which helped us to revise and improve the manuscript. Most comments seemed to be fair and reasonable, so we revise the manuscript according to their advice and suggestions. We believe that the contents and the clarity of our paper are improved in the re-submitted version. Below are point-by-point responses to the reviewers and the journal requirements comments. The revised manuscript was submitted as ‘Highlighted Revised Manuscript’ file which all the requested corrections was addressed in highlighted in yellow. The second file as a separate file labeled 'Manuscript' was also uploaded. I hope the responses satisfy you and the editorial groups in order to publish our paper.

Sincerely Yours,

Abdollah Derakhshandeh

Department of Pathobiology, School of Veterinary Medicine, Shiraz University, Shiraz, 71345-1731, Iran. Tel: +98-7136138666, Mobile: +98-9171825358, Fax: +98-7132286940, Email: [drkhshnd77@gmail.com](mailto:drkhshnd77@gmail.com).

**Journal Requirements:**

1. Please ensure that your manuscript meets PLOS ONE's style requirements, including those for file naming.

- Response: Yes, All style requirements was done.

2. Thank you for stating the following financial disclosure:

"This work was financially supported partially by a grant from Shiraz University (98GCU2M163973) and also Iran National Science Foundation (INSF) (No. 98023138). "

Please state what role the funders took in the study. If the funders had no role, please state: "The funders had no role in study design, data collection and analysis, decision to publish, or preparation of the manuscript." If this statement is not correct you must amend it as needed. Please include this amended Role of Funder statement in your cover letter; we will change the online submission form on your behalf.

- Response: The sentence was added in funding statement and highlighted in yellow

3. Thank you for stating the following in the Acknowledgments Section of your manuscript:

"This work was financially supported partially by a grant from Shiraz University (98GCU2M163973) and also Iran National Science Foundation (INSF) (No. 98023138). "

We note that you have provided funding information that is not currently declared in your Funding Statement. However, funding information should not appear in the Acknowledgments section or other areas of your manuscript. We will only publish funding information present in the Funding Statement section of the online submission form.

Please remove any funding-related text from the manuscript and let us know how you would like to update your Funding Statement. Currently, your Funding Statement reads as follows: "This work was financially supported partially by a grant from Shiraz University (98GCU2M163973) and also Iran National Science Foundation (INSF) (No. 98023138). "

Please include your amended statements within your cover letter; we will change the online submission form on your behalf.

- Response: Please include the statement "This work was financially supported partially by a grant from Shiraz University (98GCU2M163973) and also Iran National Science Foundation (INSF) (No. 98023138)." in you online submission on behalf of us. We deleted funding information in the Acknowledgments section or other areas of our manuscript.

4. Please review your reference list to ensure that it is complete and correct

- Response: All references are complete and correct.

**Reviewer Comments:**

I have reviewed the manuscript entitled  "Immunoinformatics Analysis of Candidate Proteins for Controlling Bovine Paratuberculosis" by Moezzi et. al., The authors have evaluated the efficacy of two proteins MAP2191 and FAP-P to be a potent vaccine candidate against MAP infection using immunoinformatics approaches.

- Response: Thanks for kind words and sentences about our study.

1. The authors have selected only one proteins MAP2191 out of cluster of 6 genes, why only one?, it has been mentioned that all these proteins are involved MAP invasion, survival and virulence (Hemati Z, 2018; Semret M, 2005).

- Response:

Thank you for the comment. Because of identifying and submitting the nucleotide sequence of the MAP2191 gene and some experimental works have been performed on the MAP2191 protein in our lab. So, in this study, the authors have decided to consider the MAP2191 amino acid sequence for immunoinformatics evaluations. Finally, when any researchers work on MAP, she/he must consider the cross reactivity with *Mycobacterium* *bovis.* MAP 2191shows no cross reactivity with TB.

2. The author did not mention anywhere whether these protein will act as protective antigen against all serovars or single serovars of MAP.

- Response:

Thanks for raised comments. As stated at the beginning of the results, conserved regions of these two proteins from 12 and 20 amino acid sequences of MAP2191 and FAP-P were selected, respectively. Due to the consideration of conserved regions of different sequences available in the GenBank, the authors believe these two proteins can act as protective antigens against different serovars of MAP.

3. The author should also explore the Virulence database.

- Response:

Many thanks for your suggestion. Regarding the comment of the respected reviewer, the authors have searched the virulence database (VFDB) as the reference database. Two genomes are available for MAP in this database (k10 and MAP4), and their sequences have been released in NCBI. So, the amino acid sequences of MAP2191 and FAP-P that are available in NCBI have been considered in this study.

4. The author can explore the confident B-Cell epitope by using two or more different tool and common prediction by all these will be considered confident B-cell epitope (conformational as well as linear epitope).

- Response:

Many thanks for your good comment. To comply with the reviewer's comment, two servers were considered for linear and conformational epitope prediction in the revised version of the manuscript. The results are given in Table 4 and Table 6, respectively.
